# Exploring the Landscape of Complementary and Alternative Medicine (CAM) among Multiple Sclerosis Patients in Riyadh: Types, Patterns, Health Implications, and Social Drivers – A Cross‑Sectional Study

**DOI:** 10.1007/s44446-026-00091-1

**Published:** 2026-06-13

**Authors:** Tahani M. Aljohani, Gadah A. Al-Hamoud

**Affiliations:** 1 Department of Social Studies, College of Humanities and Social Sciences, King Saud University, P.O. Box 2457, 11451 Riyadh, Saudi Arabia; 2 Department of Pharmacognosy, College of Pharmacy, King Saud University, P.O. Box 2457, 11451 Riyadh, Saudi Arabia

**Keywords:** Complementary and Alternative Medicine (CAM), Multiple Sclerosis (MS), Medical Sociology, Pharmacognosy, Medical pluralism, Health-seeking behavior

## Abstract

This cross-sectional study examines the use of complementary and alternative medicine (CAM) among patients with multiple sclerosis (MS) in Riyadh. A survey was conducted among 151 multiple sclerosis patients enrolled at the neurology clinics of two hospitals in Riyadh (KSUMC and KFMC). The study indicated that a significant majority of respondents (60.93%) used complementary and alternative medicine, demonstrating a pronounced preference for religious–spiritual modalities, including Islamic ruqyah and cupping therapy, in addition to other practices such as massage, oils, dietary supplements, honey, and herbal remedies. The findings underscore the importance of symptom alleviation, optimism about a cure or a delay in disease progression, and social sources of support (from family, healthcare professionals, and the media) in driving decisions to use CAM. The results showed an association between educational level, marital status, and CAM use. Additionally, many patients did not disclose their CAM use to their physicians or pharmacists, either because they perceived it as insignificant or because they did not receive sufficient prompting to disclose it. This highlights the need for more proactive, non‑judgmental communication from healthcare practitioners about CAM, as well as targeted training to improve patients' understanding of potential interactions between CAM and conventional treatments.

**Supplementary Information:**

The online version contains supplementary material available at 10.1007/s44446-026-00091-1.

## Introduction:

Multiple sclerosis (MS) is a chronic, progressive, and disabling neurological disorder characterized by inflammation and demyelination within the central nervous system (Compston and Coles 2008). MS affects more than 2.8 million people worldwide; the number of people with the condition varies widely across regions (MSIF 2022). Recent estimates indicate that about 1 million people in the United States have MS, with a prevalence rate of approximately 309.2 per 100,000 adults as of 2010. The disease affects women more than men (2.8 women per man) and is more common in northern regions (Wallin et al. 2019). The increase in prevalence has been partly attributed to improvements in diagnostic criteria and increased disease awareness.

Patients with multiple sclerosis experience substantial physical and mental health burdens that significantly reduce their health-related quality of life and are associated with marked functional limitations in daily activities. These challenges persist even though disease-modifying treatments (DMTs) have substantially expanded over the past decades and can reduce relapse rates and possibly slow disability progression in relapsing forms of multiple sclerosis, particularly when initiated early in the disease course (Heesen et al. 2024; Li 2022). This therapeutic gap, combined with the disease's long-term nature, leads many people with MS to seek complementary and alternative medicine (CAM) as an adjunct to, or sometimes a replacement for, conventional treatments. CAM is a broad term that encompasses a range of medical and healthcare systems, practices, and products that are not typically considered part of conventional medicine. The National Center for Complementary and Integrative Health (NCCIH) defines CAM as natural products like herbs, vitamins, minerals, and probiotics; mind–body practices like meditation, yoga, and relaxation techniques; and traditional healing systems like acupuncture, Ayurveda, and traditional Chinese medicine (NCCIH 2021). In some cultural contexts, religious and spiritual practices such as prayer and other traditional rituals are regarded as forms of CAM. In the present study, CAM was operationally defined to include the modalities that are commonly used by patients with multiple sclerosis in the Saudi context. Based on the World Health Organization’s descriptions of traditional and complementary medicine (World Health Organization 2013), which distinguish between natural products and various non‑pharmacological procedures, the modalities in this study were grouped into three broad categories: (1) religious–spiritual therapies (e.g. Islamic ruqyah and cupping therapy); (2) manual and physical therapies (e.g. massage and the use of therapeutic oils); and (3) natural product–based therapies (e.g. dietary supplements, honey, and herbal remedies). The reported prevalence of CAM use among people with MS is high, often ranging from about one-third to over two-thirds of patients, depending on the country, study design, and how CAM is defined (Lotfi et al. 2024; Olsen 2009). Patterns of CAM use vary across countries, influenced by cultural norms, religious beliefs, and the structure and accessibility of the healthcare system (Farhoudi et al. 2019; Lopez-Alcalde et al. 2025; Lotfi et al. 2024; Podlecka-Piętowska et al. 2022; Olsen 2009). In a prospective clinical study conducted at an academic MS center in the northeastern United States, approximately one-third of patients with MS (30.5%) reported using at least one form of complementary and alternative medicine, most commonly chiropractic care, massage, and acupuncture (Kim et al. 2018). Recent studies from European countries and the Middle East have reported similarly high rates of CAM use among people with MS, with patients frequently utilizing herbal products, manual therapies, mind–body practices, and religious or spiritual approaches to cope with persistent symptoms and long-standing disease, and often in the context of dissatisfaction with conventional therapies (Farhoudi et al. 2019; Lopez-Alcalde et al. 2025; Lotfi et al. 2024; Podlecka-Piętowska et al. 2022; Olsen 2009). In these diverse contexts, recurring themes include the quest for enhanced symptom management, increased well-being, and alignment with personal and cultural values, establishing CAM as a crucial, yet often under‑recognized, component of MS care experience.

According to national data from Saudi Arabia, multiple sclerosis affects about 40 out of every 100,000 in the total population, and 61.9 per 100,000 among Saudi nationals. Women are about twice as likely as men to be affected, which is consistent with global observations of female predominance in MS (AlJumah 2020). In Saudi Arabia, the use of CAM is widespread, with a large proportion of the public reporting awareness and self-use of herbs, nutritional supplements, and religious or spiritual practices such as prayer, reflecting strong cultural and religious influences on health-seeking behavior (Alrowais and Alyousefi 2017; Zaidi et al. 2022). In 2019 and 2020, studies from a multiple sclerosis clinic in Khobar and a tertiary MS center in Riyadh, respectively, demonstrated a very high prevalence of CAM use among Saudi patients with multiple sclerosis, with many individuals reporting practices such as praying, reciting the Quran, and taking vitamin supplements, while a considerable proportion did not disclose their CAM use to their physicians (Alnahdi et al. 2020; Shariff et al. 2019). More recently, a 2024 cross-sectional study from Saudi Arabia confirmed that CAM remains commonly used among people living with MS, with vitamins and other nutritional supplements among the most frequently reported modalities and limited patient–physician communication about CAM use still being a concern (Aljarallah et al. 2024). Nevertheless, data specifically examining complementary and alternative medicine (CAM) utilization among multiple sclerosis patients in Saudi Arabia are scarce, particularly regarding the specific types of therapies employed, perceived health effects—both beneficial and detrimental—patterns of disclosure to various healthcare providers, and the influence of demographic and social factors on these patterns. Sociology is particularly well-suited to elucidating the impact of social forces on individual health behaviors, including the utilization of CAM (Stratton and McGivern-Snofsky 2008). Although several Saudi studies have documented the prevalence and general patterns of CAM use among patients with MS, they have primarily focused on describing overall use and commonly employed modalities, with limited attention to the social drivers, disclosure behaviors, and multi-dimensional reasons underlying CAM use in this population (Alnahdi et al. 2020; Shariff et al. 2019; Aljarallah et al. 2024). In contrast, the present study not only estimates the prevalence and types of CAM used by patients with MS in Riyadh but also employs a structured, multi-dimensional scale to examine illness-related, social environment–related, knowledge-related, safety-related, and treatment-related reasons for CAM use. In addition, the multi-dimensional reasons-for-use scale developed for this study demonstrated acceptable face and content validity and internal consistency reliability, although more comprehensive psychometric validation, including assessment of construct validity, is still needed. Furthermore, the study provides a detailed analysis of disclosure to physicians and pharmacists and the reasons for non-disclosure, thereby offering a more comprehensive, sociologically informed understanding of CAM use in this context. Consequently, this cross-sectional study aims to delineate the utilization of CAM among patients with MS by estimating its prevalence, examining the influence of demographic and sociological determinants, and analysing multi-dimensional reasons for CAM use and patterns of disclosure to healthcare providers.

## Methods

### Study design and setting

This cross-sectional study was conducted using a structured, self-administered online questionnaire between June and November 2025. Eligible participants were Saudi patients of both sexes with a documented diagnosis of MS, aged ≥ 18 years, who had regular follow-up appointments at the participating neurology clinics, had sufficient physical and cognitive ability to read and complete the questionnaire, and provided consent to participate. Patients with severe cognitive impairment or an acute relapse that precluded completion of the questionnaire were excluded. The target population, therefore, comprised adult MS patients who were registered and followed at the neurology clinics of King Saud University Medical City (KSUMC) and King Fahad Medical City (KFMC) in Riyadh. All patients had an established MS diagnosis documented by their treating neurologists at KSUMC or KFMC and were attending scheduled follow-up visits during the study period. When inviting patients to participate, the research team verbally confirmed that they had been diagnosed with MS and were being followed in these neurology clinics, to ensure that only eligible patients completed the questionnaire. Participation was voluntary, and all participants provided informed consent before completing the questionnaire. No formal a priori sample size calculation was conducted; instead, all eligible patients attending the participating clinics during the study period were invited to participate. Given the exploratory nature of the study, the sample size was determined pragmatically by the number of eligible patients available. A total of 155 patients agreed to participate, and 151 completed the questionnaire with sufficient data for analysis, yielding a completion rate of 97.9% and a final analysed sample of 151 MS patients.

### Survey instrument development, validation, and pilot study

Data were collected using a structured online questionnaire, developed specifically for this study, to capture sociodemographic characteristics, patterns of CAM use, perceived health effects, disclosure behaviors, and reasons for CAM use among MS patients. The questionnaire was originally developed in Arabic, the native language of the target population, to ensure clarity and cultural relevance, and consisted of four main sections. The first section collected sociodemographic information, including sex, age, educational level, and marital status. The second section assessed the extent of CAM use, the types of CAM modalities utilized, and respondents’ perceptions of the benefits and adverse effects associated with their use. The list of CAM modalities included in the questionnaire was developed based on prior literature on CAM use among patients with multiple sclerosis in Saudi Arabia and other countries, together with local clinical experience [Alnahdi et al. 2020; Shariff et al. 2019; Aljarallah et al. 2024; Kim et al. 2018; Lotfi et al. 2024]. For conceptual purposes and drawing on the World Health Organization’s descriptions of traditional and complementary medicine, CAM was classified into three broad categories: religious–spiritual therapies, manual/physical therapies, and natural product–based therapies (World Health Organization 2013). In the questionnaire and subsequent analysis, these broad categories were further operationalised into specific CAM categories: manipulative and body‑based practices, biologically based practices (including dietary or nutrition‑based therapies), mind‑body medicine, spiritual or faith‑based therapies, creative therapies, and others as presented in Table 11. The third section explored the extent to which healthcare providers were informed about CAM use and the reasons for non-disclosure among respondents who did not notify their providers. The fourth section comprised a multi-dimensional scale measuring reasons for CAM use among MS patients from the respondents’ perspective. All participants, including those who had previously used CAM and those who had not, were asked to indicate their level of agreement with statements describing potential reasons that might lead people with MS to use CAM. The items of the multi-dimensional “reasons for CAM use” scale were generated through an extensive review of international and local literature on CAM use among patients with chronic conditions and MS, supplemented by input from pharmacists and sociologists with expertise in MS and health-seeking behaviour (Tan et al. 2013; Lopez-Alcalde et al. 2025; Lotfi et al. 2024; Alrowais and Alyousefi 2017). This process yielded an initial pool of 22 statements representing potential motivations for CAM use, and draft items were refined to ensure comprehensive coverage of illness-related, treatment-related, safety-related, social environment–related, and knowledge-related reasons. The items were grouped into five dimensions: (1) belief in the safety of folk and alternative treatments (five items); (2) treatment-related factors (three items); (3) illness-related factors (three items); (4) social environment factors (seven items); and (5) cognitive factors (four items). This grouping into five conceptual dimensions was carried out by the research team after generating and refining the item pool to reflect distinct domains of potential reasons for CAM use (safety-related, illness-related, treatment-related, social environment-related, and knowledge-related factors). Each item was rated on a 5-point Likert scale ranging from 1 “strongly disagree” to 5 “strongly agree”, and dimension-specific mean scores were calculated by averaging the items within each dimension, with higher scores indicating stronger endorsement of the corresponding reasons. An overall mean score for the full scale was also computed to reflect the general strength of endorsement of reasons for CAM use. In the Results and Discussion sections, higher mean scores within a given dimension are interpreted as indicating that these reasons played a more prominent perceived role in motivating CAM use among patients with MS.

Face and content validity of the questionnaire, including the reasons-for-use scale, were assessed by an expert panel of three faculty members in sociology and two pharmacists, who independently evaluated each item for clarity, relevance, and representativeness of the underlying construct. Minor wording changes were made based on their feedback, and the revised version was then piloted on a convenience sample of 30 individuals to assess comprehensibility and feasibility. Participants reported that the items were easy to understand, and only minor wording adjustments were required. Internal consistency of the multi-dimensional reasons-for-use scale was evaluated using Cronbach’s alpha, with all dimensions exceeding the commonly accepted threshold of 0.70 (Nunnally and Bernstein 1994), indicating acceptable to good internal consistency reliability. These procedures provided initial evidence of face and content validity and internal consistency for the multi-dimensional reasons-for-use scale; however, more extensive psychometric evaluation (e.g. factor analysis or assessment of construct validity) was beyond the scope of this study. The alpha values were 0.879 for the first dimension, 0.754 for the second, 0.862 for the third, 0.973 for the fourth, and 0.863 for the fifth, while the overall Cronbach’s alpha for the full scale was 0.952.

### Statistical analysis

Data were entered and analysed using IBM SPSS Statistics software. Descriptive statistics, including frequencies, percentages, means, and standard deviations, were used to summarise sociodemographic characteristics, patterns of CAM use, perceived health effects, disclosure behaviors, and reasons for CAM use. Associations between categorical variables were examined using the chi-square test, and associations were considered statistically significant at p < 0.05. For continuous quantitative data on participants’ perceptions of reasons for CAM use, mean scores and standard deviations were calculated, and the reasons were ranked in descending order by their mean values. Differences in responses regarding reasons for CAM use between participants who had previously used CAM and those who had not were assessed using independent-samples t-tests, with p < 0.05 set as the criterion for statistical significance. Given the exploratory and descriptive nature of the study and the available sample size, we primarily relied on bivariate analyses (chi-square tests and independent-samples t-tests) to examine associations between CAM use and sociodemographic variables. Multivariable models were considered but not implemented because the number of outcome events and the distribution of covariates were judged insufficient to support a stable logistic regression model with several predictors.

## Results

This section is organised into four parts. The first describes the sociodemographic characteristics of the study sample; the second examines the prevalence of CAM use among participants and its association with their sociodemographic characteristics; the third presents participants’ perceptions of the reasons for their CAM use and compares responses between those who had used CAM and those who had not. The fourth part, restricted to patients who had previously used CAM, focuses on the most commonly used CAM modalities and the perceived impact of CAM use in achieving the desired benefit or experiencing adverse effects. In addition, this section reports the extent to which patients disclosed their CAM use to healthcare providers and the reasons for non-disclosure among those who did not.

### Sociodemographic characteristics

The study sample comprised 151 patients with MS attending neurology clinics at KSUMC and KFMC. Table 1 presents the sociodemographic characteristics of the participants. Females represented 76.82% of the sample, whereas males accounted for 23.18%. The mean age was 36.01 years, with the largest proportion in the 31–40-year age group, and most participants held a bachelor’s degree (60.93%). Nearly half of the sample were married (48.34%).

**Table 1:** Sociodemographic characteristics of the study sample (n = 151)

n (%)			Category	Variable
(23.18)	35		Male	**Sex**
(76.82)	116		Female	
**(100.0)**	**151**		**Total**	
(2.65)	4		20—18	**Age (years)**
(29.14)	44		30—21	
(39.07)	59		40—31	
(19.21)	29		50—41	
(7.95)	12		60—51	
(1.32)	2		60 $<$	
(0.66)	1		Missing	
36.0 $\pm 9.97$			Mean ($\pm \mathrm{S}\mathrm{D})$	
**(100.0)**		**151**	**Total**	
(16.55)		25	High school or less	**Educational level**
(11.26)		17	Diploma	
(60.93)		92	Bachelor’s	
(8.61)		13	Master’s	
(2.65)		4	Doctorate	
**(100.0)**		**151**	**Total**	
(37.75)		57	Single	**Marital status**
(48.34)		73	Married	
(13.91)		21	Divorced	
**(100.0)**		**151**	**Total**	

### Prevalence of CAM use and its association with sociodemographic characteristics

Among the 151 MS patients, 60.93% reported prior use of CAM, whereas 39.07% had never used it (Table 2). Chi-square tests (Table 3) showed no significant association between sex and CAM use (χ^2^ = 0.44, p = 0.508) and no significant association between age and CAM use (χ^2^ = 2.14, p = 0.830). In contrast, there was a statistically significant association between educational level and CAM use (χ^2^ = 10.36, p = 0.035), as well as between marital status and CAM use (χ^2^ = 7.08, p = 0.029). CAM use was more frequent among participants with a bachelor’s degree or higher and among married patients compared with their counterparts.

**Table 2:** Responses of MS patients regarding prior use of CAM (n = 151)

(%)	n	Response	Item
(60.93)	92	Yes	**Since being diagnosed with MS, have you ever tried any form of CAM?**
(39.07)	59	No	
**(100.0)**	**151**	**Total**

**Table 3:** Association between sociodemographic characteristics and CAM use among MS (n = 151)

Sociodemographic variable		CAM use—Yes		CAM use—No		Total	
		**n**	**(%)**	**n**	**(%)**	**n**	**(%)**
**Sex**	Male	23	(15.23)	12	(7.95)	35	(23.18)
	Female	69	(45.70)	47	(31.13)	116	(76.82)
	**Total**	**92**	**(60.93)**	**59**	**(39.07)**	**151**	**(100.0)**
	Chi-square test for sex: χ^2^ = 0.44, *p* = 0.508 (not statistically significant)						
**Age (year)**	18—20	3	(2.00)	1	(0.67)	4	(2.67)
	21—30	24	(16.00)	20	(13.33)	44	(29.33)
	31—40	36	(24.00)	23	(15.33)	59	(39.33)
	41—50	20	(13.33)	9	(6.00)	29	(19.33)
	51—60	8	(5.33)	4	(2.67)	12	(8.00)
	60 $<$	1	(0.67)	1	(0.67)	2	(1.33)
	**Total**	**92**	**(61.33)**	**58**	**(38.67)**	**151**	**(100.0)**
	Chi-square test for age: χ^2^ = 2.14, *p* = 0.830 (not statistically significant)						
**Educational Level**	High school or less	20	(13.25)	5	(3.31)	25	(16.56)
	Diploma	13	(8.61)	4	(2.65)	17	(11.26)
	Bachelor’s	49	(32.45)	43	(28.48)	92	(60.93)
	Master’s	9	(5.96)	4	(2.65)	13	(8.61)
	Doctorate	1	(0.66)	3	(1.99)	4	(2.65)
	**Total**	**92**	**(60.93)**	**59**	**(39.07)**	**151**	**(100.0)**
	Chi-square test for educational level: χ^2^ = 10.36, *p* = 0.035 (statistically significant at the 0.05 level)						
**Marital status**	Single	30	(19.87)	27	(17.88)	57	(37.75)
	Married	44	(29.14)	29	(19.21)	73	(48.34)
	Divorced	18	(11.92)	3	(1.99)	21	(13.91)
	**Total**	**92**	**(60.93)**	**59**	**(39.07)**	**151**	**(100.0)**
	Chi-square test for marital status: χ^2^ = 7.08, *p* = 0.029 (statistically significant at the 0.05 level)						

### Patients’ perceptions of reasons for CAM use

Table 4 presents MS patients’ perceptions of reasons that might lead people with MS to use CAM, grouped by the thematic dimensions of the reasons-for-use scale. Responses were ordered by calculating mean scores and standard deviations for each item, thereby identifying the most prominent reasons participants perceived.

**Table 4:** Mean scores of thematic dimensions of reasons for CAM use among MS patients

Rank	SD	Mean	Dimension	No
4	**0.86**	**2.88**	Belief in the safety of CAM	**1**
5	**0.83**	**2.85**	Treatment-related factors (conventional medical care)	**2**
1	**1.15**	**3.61**	Illness-related factors	**3**
2	**1.03**	**2.98**	Social environment factors	**4**
3	**0.95**	**2.89**	Cognitive factors (knowledge and understanding)	**5**

At the thematic level, illness-related factors emerged as the most prominent reasons for CAM use, with a mean score of 3.61 and a standard deviation of 1.15. This was followed by social environment factors (mean 2.98, SD 1.03) and cognitive factors related to knowledge and understanding (mean 2.89, SD 0.95). Reasons related to belief in the safety of folk and alternative treatments showed a very similar level of endorsement (mean 2.88, SD 0.86), while treatment-related factors associated with conventional medical care ranked last, with a mean score of 2.85 and a standard deviation of 0.83.

#### Belief in the safety of CAM

Within the dimension of belief in the safety of CAM (Table 5), participants most frequently endorsed, as potential reasons for CAM use, the perception that complementary and alternative treatments are free of harmful drugs, with a mean score of 3.03 and a standard deviation of 1.13. The following most highly rated reason was the belief that there is no conflict between prescribed medications and CAM (mean 2.93, SD 1.10). Participants also commonly perceived CAM therapies as completely safe when used alongside prescribed medications (mean 2.88, SD1.03).

**Table 5:** Item rankings within the “belief in the safety of CAM” dimension

Statement	Strongly agree		Agree		Somewhat agree		Disagree		Strongly disagree		Mean	SD	Rank
	n	(%)	n	(%)	n	(%)	n	(%)	n	(%)			
CAM treatments are free from harmful drugs	15	(9.93)	35	(23.18)	57	(37.75)	27	(17.88)	17	(11.26)	3.03	1.13	1
There is no conflict between prescribed medications and CAM	12	(7.95)	34	(22.52)	53	(35.10)	36	(23.84)	16	(10.60)	2.93	1.10	2
CAM treatments are entirely safe when used together with prescribed medications	9	(5.96)	32	(21.19)	55	(36.42)	42	(27.82)	13	(8.61)	2.88	1.03	3
CAM treatments can be used without consulting a physician	12	(7.95)	31	(20.53)	47	(31.12)	39	(25.83)	22	(14.57)	2.81	1.16	4
CAM treatments enhance the effectiveness of prescribed medications	8	(5.30)	29	(19.20)	47	(31.12)	50	(33.11)	17	(11.26)	2.74	1.06	5

#### Treatment-related reasons for CAM use

In the treatment-related dimension (Table 6), the primary endorsed treatment-related reason that might lead MS patients to use CAM was the desire to alleviate the adverse effects of conventional medical treatment, yielding a mean score of 3.11 and a standard deviation of 1.07. The second most frequently endorsed reason was the perception that traditional treatment alone is not sufficient to address the patient’s condition (mean 2.97, SD 1.13). The lowest-ranked reason within this dimension was the perception that conventional medical treatment is not helpful for the patient’s condition (mean 2.48, SD of 1.09).

**Table 6:** Item rankings within the “treatment-related factors” dimension

Statement	Strongly agree		Agree		Somewhat agree		Disagree		Strongly disagree		Mean	SD	Rank
	n	(%)	n	(%)	n	(%)	n	(%)	n	(%)			
Desire to reduce the side effects of conventional medical treatment	13	(8.61)	41	(27.15)	61	(40.40)	21	(13.91)	15	(9.93)	3.11	1.07	1
Feeling that conventional medical treatment alone is not sufficient for the patient’s condition	16	(10.60)	28	(18.54)	59	(39.07)	31	(20.53)	17	(11.26)	2.97	1.13	2
Feeling that conventional medical treatment is not helpful for the patient’s condition	6	(3.97)	19	(12.58)	50	(33.11)	42	(27.82)	34	(22.52)	2.48	1.09	3

#### Illness-related reasons for CAM use

The main illness-related reason endorsed by people who used CAM in the illness-related component (Table 7) was the desire to relieve pain, with a mean score of 3.75 and a standard deviation of 1.17. The second most important reason was the desire to get rid of the condition (mean 3.64, SD 1.33). The third most important reason was the desire to slow the disease's progression (mean 3.46, SD 1.28).

**Table 7:** Item rankings within the “illness-related factors” dimension

Statement	Strongly agree		Agree		Somewhat agree		Disagree		Strongly disagree		Mean	SD	Rank
	no	(%)	no	(%)	no	(%)	no	(%)	no	(%)			
Patient’s desire to relieve pain	52	(34.44)	39	(25.83)	37	(24.50)	16	(10.60)	7	(4.63)	3.75	1.17	1
The patient’s desire to get rid of the disease	55	(36.43)	31	(20.53)	35	(23.18)	15	(9.93)	15	(9.93)	3.64	1.33	2
Patient’s desire to slow disease progression	40	(26.49)	38	(25.17)	39	(25.83)	19	(12.58)	15	(9.93)	3.46	1.28	3

#### Social environment–related reasons for CAM use

Within the social environment dimension, the most common endorsed reason that might lead MS patients to use CAM was being advised to do so by a pharmacist, as shown in Table 8, with a mean score of 3.24 and a standard deviation of 1.19. The second most common reason was using CAM because a relative recommended it (mean 3.01, SD 1.14). The third most common reason was using CAM on the advice of an acquaintance (e.g., coworker or neighbor) (mean 2.97, SD 1.13).

**Table 8:** Item rankings within the “social environment factors” dimension

Statement	Strongly agree		Agree		Somewhat agree		Disagree		Strongly disagree		Mean	SD	Rank
	n	(%)	n	(%)	n	(%)	n	(%)	n	(%)			
The patient uses CAM if advised to do so by a pharmacist	25	(16.56)	39	(25.82)	48	(31.79)	25	(16.56)	14	(9.27)	3.24	1.19	1
The patient uses CAM if advised to do so by a relative	19	(12.58)	26	(17.22)	60	(39.73)	30	(19.87)	16	(10.60)	3.01	1.14	2
The patient uses CAM if advised to do so by an acquaintance (e.g., work colleague or neighbour)	16	(10.60)	29	(19.2)	57	(37.75)	32	(21.19)	17	(11.26)	2.97	1.13	3
The patient uses CAM if advised to do so by a family member	16	(10.60)	25	(16.56)	60	(39.73)	35	(23.18)	15	(9.93)	2.95	1.11	4
The patient uses CAM at the advice of a friend	16	(10.60)	26	(17.22)	59	(39.07)	34	(22.51)	16	(10.60)	2.95	1.12	5
The marketing of CAM products in some shops encourages patients to use them	12	(7.95)	37	(24.50)	49	(32.45)	33	(21.85)	20	(13.25)	2.92	1.15	6
The patient uses CAM if advised to do so by another person with multiple sclerosis	13	(8.61)	24	(15.90)	58	(38.41)	41	(27.15)	15	(9.93)	2.86	1.08	7

#### Knowledge-related reasons for CAM use

In the knowledge-related dimension (Table 9), the most commonly endorsed factor that might encourage CAM use was exposure to television programs discussing the benefits of complementary and alternative medicine (mean 3.15, SD 1.12). The second most common reason was reading about the benefits of specific CAM modalities on websites (mean 2.96, SD 1.18). The third factor in this domain was information from social media—such as posts or discussions by influencers about the benefits of certain CAM modalities—which increased patients’ likelihood of using them (mean 2.87, SD 1.13).

**Table 9:** Item rankings within the “knowledge-related factors” dimension

Statement	Strongly agree		Agree		Somewhat agree		Disagree		Strongly disagree		Mean	SD	Rank
	n	(%)	n	(%)	n	(%)	n	(%)	n	(%)			
Television programmes that present the benefits of CAM encourage patients to use it	20	(13.24)	35	(23.18)	56	(37.09)	28	(18.54)	12	(7.95)	3.15	1.12	1
Reading about the benefits of a specific type of CAM on websites encourages patients to use it	18	(11.92)	28	(18.54)	55	(36.42)	30	(19.87)	20	(13.25)	2.96	1.18	2
When a social media influencer discusses the benefits of a specific CAM, it encourages patients to use it	15	(9.93)	24	(15.89)	56	(37.09)	38	(25.17)	18	(11.92)	2.87	1.13	3
Reading about CAM treatments in specialised scientific books is one of the reasons patients use them	7	(4.63)	26	(17.22)	47	(31.13)	37	(24.50)	34	(22.52)	2.57	1.15	4

### Differences in reasons according to CAM use

Participants’ responses regarding perceptions of reasons for CAM use were compared between those who had previously used CAM and those who had not, using independent-samples t‑tests. The results (Table 10) showed statistically significant differences between the two groups in the dimensions of belief in the safety of CAM and treatment-related factors at the 0.01 level, as well as in the overall mean score for the reasons-for-use scale at the 0.05 level. These differences were in favor of participants who had used CAM, indicating that prior CAM users expressed stronger agreement with the reasons that might lead MS patients to use CAM in general, and particularly with reasons related to perceived safety and to issues surrounding conventional medical treatment.

**Table 10:** Differences in mean scores on dimensions of reasons for using complementary and alternative medicine among multiple sclerosis patients according to prior CAM use

Dimension	Have you ever tried any type of CAM?	n	Mean	SD	df	t	P value
Dimension 1: Belief in the safety of complementary and alternative medicine	Yes	92	3.07	0.84	149	3.59	0.000**
	No	59	2.58	0.82			
Dimension 2: Treatment-related factors	Yes	92	2.99	0.81	149	2.61	0.009**
	No	59	2.63	0.82			
Dimension 3: Illness-related factors	Yes	92	3.68	1.08	149	0.85	0.397
	No	59	3.51	1.26			
Dimension 4: Social environment–related factors	Yes	92	3.10	1.06	149	1.71	0.089
	No	59	2.81	0.97			
Dimension 5: Knowledge-related factors	Yes	92	2.95	0.95	149	0.94	0.350
	No	59	2.80	0.95			
Overall mean score of reasons that may lead multiple sclerosis patients to use CAM	Yes	92	3.13	0.73	149	2.48	0.014*
	No	59	2.83	0.75			

^*^Statistically significant at the 0.05 level

^**^Statistically significant at the 0.01 level

In contrast, no statistically significant differences were observed between CAM users and non‑users in the illness-related, social environment–related, or knowledge-related dimensions of the scale. This suggests that, regardless of their own CAM use, participants reported broadly similar views about reasons related to the nature of the disease itself, the influence of the social environment, and informational or knowledge-based factors.

### Types of complementary and alternative medicine used by the respondents and the extent of positive and negative effects resulting from their use

Table 11 presents the responses of multiple sclerosis patients who had previously used CAM (n = 92 out of the total study sample) regarding the types of CAM they had used, classified according to the World Health Organization’s categorization. Among CAM users, the most commonly used type was spiritual healing through Islamic ruqyah, accounting for 38.41% of reported use, followed by cupping therapy at 34.44%. Use of oils and massage each reached 19.87%, followed by dietary supplements at 18.54%, and finally herbal remedies and honey, each at 16.56%. Taken together, these results indicate that religious–spiritual therapies rank highest in use among the respondents, followed by manual/physical therapies, and then natural product–based therapies.

**Table 11:** Types of CAM used by respondents with MS

CAM category	Therapy	n	(%)	Rank
Question Have you ever informed your physician about your use of complementary and alternative medicine (CAM)?	**Cupping therapy**	52	(34.44)	2
	**Massage**	30	(19.87)	3
	**Acupuncture**	11	(7.28)	7
	**Cauterization**	11	(7.28)	7
If you did not inform your physician, what was the reason?	**Dietary supplements (without a physician’s prescription)**	28	(18.54)	4
	**Herbal remedies**	25	(16.56)	5
	**Honey therapy**	25	(16.56)	5
	**Bee venom therapy**	14	(9.27)	6
	**Traditional mixtures prepared by herbalists**	14	(9.27)	6
	**Oils**	30	(19.87)	3
Have you ever informed the pharmacist about your use of complementary and alternative medicine (CAM)?	**Plant-based (vegetarian) diet**	9	(5.96)	10
	**Ketogenic diet**	6	(3.97)	9
	**Gluten-free diet**	5	(3.31)	11
	**Meditation**	8	(5.30)	8
	**Yoga**	11	(7.28)	7
If you did not inform the pharmacist, what was the reason?	**Islamic ruqyah**	58	(38.41)	1
	**Art therapy**	2	(1.32)	12
	**Music therapy**	1	(0.66)	13
		340*	(100.0)	
	**Other CAM type**			
* Percentages in this table are calculated among CAM users only (n = 92).	1		(0.66)	
** Total n for this section includes only participants who answered "No" or "Sometimes" to the preceding question.	1		(0.66)	
Question	1		(0.66)	
Have you ever informed your physician about your use of complementary and alternative medicine (CAM)?	1		(0.66)	

The number of respondents was 92; however, participants were allowed to select more than one option for this question

### Perceived benefits and adverse effects associated with CAM use among users

Among the 92 participants who had previously used complementary and alternative medicine (CAM), responses reflected the extent to which they obtained the expected benefits and experienced adverse effects (Table 12). Regarding the expected benefit from CAM use, 44.56% reported obtaining the anticipated benefit several times, 19.56% once, and 35.86% indicated they had not obtained it. Regarding adverse effects associated with CAM use, 73.91% reported never experiencing any adverse effects, 19.56% once, and 6.52% several times.

**Table 12:** Extent to which MS patients achieve expected benefits and experience adverse effects from CAM use

Question	Category	n	(%)
Have you obtained the expected benefit from your use of complementary and alternative medicine (CAM)?	Yes, several times	41	(44.56)
	Yes, once	18	(19.56)
	No, this has not happened before	33	(35.86)
	Total	92*	(100.0)
Have you ever experienced adverse effects as a result of using complementary and alternative medicine (CAM)?	Yes, several times	6	(6.52)
	Yes, once	18	(19.56)
	No, this has not happened before	68	(73.91)
	Total	92*	(100.0)

^*^The total number here represents only the participants who had used complementary and alternative medicine, excluding those who had never used it

### Awareness of healthcare providers about CAM use

Table 13 presents the responses of participants who had used complementary and alternative medicine (CAM) regarding whether they informed their healthcare providers about their CAM use and their reasons for not doing so. Among CAM users (n = 92), 58.69% of respondents reported that their doctor was not aware of their use of CAM, whereas 30.43% stated that they informed their doctor, and 10.86% reported that they informed their doctor only sometimes. Regarding reasons for not informing the physician, 51.56% of participants indicated that they thought it was not important to mention CAM use to their doctor, 31.81% stated that the doctor did not ask, and 12.50% reported being afraid of being criticized.

**Table 13:** Extent of informing healthcare providers about the use of complementary and alternative medicine and reasons for non‑disclosure among CAM users (n = 92)

Question	Category	n	(%)
Have you ever informed your physician about your use of complementary and alternative medicine (CAM)?	Yes	28	(30.43)
	No	54	(58.69)
	Sometimes	10	(10.86)
	Total	92*	(100.0)
If you did not inform your physician, what was the reason?	The physician did not ask	21	(32.81)
	I did not think it was important	33	(51.56)
	I was afraid of criticism	8	(12.50)
	No response	2	(3.12)
	Total	64**	(100.0)
Have you ever informed the pharmacist about your use of complementary and alternative medicine (CAM)?	Yes	11	(11.95)
	No	76	(82.60)
	Sometimes	3	(3.26)
	No response	2	(2.17)
	Total	92*	(100.0)
If you did not inform the pharmacist, what was the reason?	The pharmacist did not ask	33	(41.77)
	I did not think it was important	41	(51.89)
	I was afraid of criticism	2	(2.53)
	No response	3	(3.79)
	Total	79**	(100.0)

^*^ Percentages in this table are calculated among CAM users only (n = 92)

^**^ Total n for this section includes only participants who answered “No” or “Sometimes” to the preceding question

Regarding pharmacist disclosure, among CAM users (n = 92), 82.60% reported not informing the pharmacist about their CAM use, 11.95% reported informing the pharmacist, and 3.26% reported sometimes informing the pharmacist. Regarding reasons for not informing the pharmacist, 51.89% indicated that they did not think it was important to mention CAM use to the pharmacist, 41.77% reported that the pharmacist did not ask them, and 2.53% stated that they were afraid of being criticized.

## Discussion

The findings of the present study indicate that the majority of participants had previously used complementary and alternative medicine (CAM), with a prevalence of 60.93% in the study sample. This level of use is broadly consistent with earlier reports showing that CAM is commonly used by people with multiple sclerosis in different settings (AlJumah et al. 2020; Alrowais and Alyousefi 2017; Farhoudi et al 2019; Kim et al. 2018; Lopez-Alcalde et al. 2025; Lotfi et al. 2024; Podlecka-Piętowska et al. 2022; Olsen 2009). In our sample, women were more prevalent than men. This predominance of female participants is consistent with national registry data from Saudi Arabia, where approximately two-thirds of MS patients are female, and the overall female-to-male ratio is about 2:1. The current study also found no statistically significant association between gender or age and CAM use, whereas significant associations were observed between CAM use and both educational level and marital status. This finding is in line with previous studies that reported a higher tendency to use CAM among patients with higher educational attainment, as well as differences in CAM-related attitudes across marital status categories (AlJumah et al. 2020; Alnahdi et al. 2020; Alrowais and Alyousefi 2017; Shariff et al. 2019).

Regarding CAM types, the most commonly reported modality in the present study was Islamic ruqyah (Quran-based spiritual healing), at 38.41%, followed by cupping therapy at 34.44%. With a considerable gap, massage and oils ranked third (19.87%), dietary supplements without a physician’s prescription ranked fourth (18.54%), and honey and herbal remedies were each reported by 16.56% of participants. These results are broadly consistent with earlier studies in which cupping therapy and religious practices were among the most commonly used CAM modalities among patients with MS across different settings (Lotfi et al. 2024; Shariff et al. 2019). However, the present study differs in that Islamic ruqyah emerged as the most frequently used modality, ranking higher than cupping therapy.

In terms of perceived benefits and adverse effects, 44.56% of participants reported that they had obtained the expected benefit from CAM use on several occasions, whereas 35.86% stated that they had not obtained the had anticipated benefit. Overall, these self‑reported perceptions suggest that many users found CAM beneficial; however, these findings are based on subjective reports and do not provide direct evidence of clinical effectiveness. This pattern is broadly consistent with findings from Morocco, where 66.3% of respondents with MS reported that alternative medicine had a positive effect on their health (Lotfi et al. 2024).

Our study found that, regarding adverse effects, most participants (73.91%) reported never experiencing side effects from CAM use, whereas 19.56% reported experiencing adverse effects only once. The high proportion of participants who reported no adverse effects suggests that many did not experience obvious harms; nevertheless, these self-reports cannot exclude unrecognised adverse effects or interactions. Regarding disclosure of CAM use to healthcare providers, the present study examined this aspect and addressed a knowledge gap concerning reasons for non-disclosure among MS patients. Overall, 58.69% of participants reported that they had never informed their physician about their CAM use, 30.43% stated that they had informed their physician, and 10.86% reported that they sometimes did so.

Concerning reasons for non-disclosure to physicians, 51.56% indicated that they believed it was not important to mention CAM use, 32.81% reported that the physician did not ask, and 12.5% stated that they were afraid of criticism. Regarding pharmacists, 82.60% of participants reported that they had never informed the pharmacist about their CAM use, compared with 11.95% who reported that they did inform the pharmacist and 3.26% who sometimes did so. As for reasons for not informing the pharmacist, 51.89% stated that they did not think it was important, 41.77% reported that the pharmacist did not ask them, and 2.53% indicated that they were afraid of criticism.

These findings suggest that participants were more inclined to disclose their CAM use to physicians than to pharmacists and highlight the importance of educating patients to inform their physicians about CAM use, as this may help reduce the risk of therapeutic conflicts and adverse effects. These findings suggest that members of the healthcare team, including physicians and pharmacists, may benefit from routinely asking patients about their use of CAM, as this is part of their professional responsibility, particularly given that many patients, as shown by the present findings, rely on physician-initiated inquiries. Furthermore, the higher proportion of participants who feared criticism from physicians compared with pharmacists highlights the need to encourage physicians to reassure patients that questions about CAM use are intended to ensure safe and effective care rather than to pass judgment.

The study also addressed the gap related to reasons for CAM use by asking both CAM users and non-users about their views regarding the factors that might lead MS patients to use CAM. These reasons were grouped into five dimensions, and the ranking of individual reasons within each dimension was presented in the results section. The dimensions were ordered as follows: first, illness-related reasons; second, social environment–related reasons; third, knowledge-related reasons; fourth, reasons related to beliefs about the safety of CAM; and fifth, reasons related to conventional medical treatment.

The present study helps fill a knowledge gap in this area by clarifying the role of the social environment in driving CAM use, given its importance in patients’ decision-making processes. Nouwens et al. (2025) noted that actual treatment decisions among patients often rely on deliberation or intuition through discussions and consultations with others (Nouwens et al. 2025). In line with this, the current study found that social environment–related reasons ranked second in their influence on CAM use, indicating their importance and strong impact. The study also identified the relative influence of different social actors in the patients’ environment on decisions to use CAM.

Based on these findings, the study suggests that implementing community-based awareness initiatives through primary healthcare centres, mass media, and social media platforms may be beneficial to provide guidance on the appropriate use of CAM in ways that do not conflict with conventional medicine and to emphasize how to verify the reliability of CAM providers. Across all dimensions, the main perceived reasons that motivated MS patients to use CAM, based on mean scores and in descending order, were as follows: the desire to relieve pain, which is consistent with previous research indicating that pain relief is a key motivation for CAM use among patients with chronic pain (Tan et al 2013); the desire to get rid of the disease; the desire to slow disease progression; using CAM when recommended by a pharmacist; exposure to television programs presenting the benefits of CAM; the desire to reduce the side effects of conventional treatment; the belief that CAM therapies are free from harmful drugs; using CAM when recommended by a relative; exposure to online information highlighting the benefits of specific CAM modalities; and finally, the belief that CAM therapies are completely safe when used alongside prescribed medications.

Taken together, these results suggest that many of the predominant perceived reasons for CAM use revolve around the desire to manage disease symptoms, attempt to eliminate the disease, or slow its progression, and this is consistent with previous findings indicating that one of the main reasons patients use CAM is to alleviate symptoms (Lopez-Alcalde et al. 2025; Nouwens et al. 2025). From a sociological perspective, this finding can be interpreted as reflecting that the disease's ambiguous nature and the unpredictability of its symptoms generate numerous questions for patients about its causes and the possibility of definitive treatment. In the absence of clear explanations or conclusive solutions within the conventional healthcare system, patients may be driven to seek answers within complementary and alternative medicine; this form of medical pluralism can be understood as an attempt to identify underlying causes and achieve effective treatment, or at least to gain better control over the disease when conventional medicine fails to address these uncertainties.

Our study also indicates that patients tend to use CAM when it is endorsed by sources they perceive as trustworthy; for example, a pharmacist's recommendation ranked fourth among the reasons participants cited. In this context, the findings of previous research are noteworthy, as most participants in those studies acknowledged the important role of pharmacists in guiding CAM use, although more than half disagreed that pharmacists were more knowledgeable in this field than other healthcare providers (Hijazi et al. 2021). Based on these findings, the present study suggests that providing appropriate training for pharmacists in the use of CAM could help them provide accurate, evidence‑based information to patients. These recommendations are in line with previous studies from Saudi Arabia and the wider region, which have consistently reported gaps in community pharmacists’ knowledge and training regarding herbal products and CAM, despite their positive attitudes and central role in guiding patients’ use of these therapies (Alsayari et al. 2018; Al-Yousf et al.2025; Jalil et al. 2022).

Furthermore, the current study showed that television programs and websites were among the most influential knowledge‑related sources motivating CAM use, surpassing, for example, social media platforms. Participants also considered relatives to be among the most influential personal factors encouraging CAM use, indicating that family members are particularly impactful in this context. Finally, patients’ belief in the safety of CAM and its lack of interaction with conventional treatment ranked only tenth among the reasons, which is broadly consistent with previous work showing that many patients perceive manual and complementary therapies as safe; this underscores the importance of educating patients about the potential adverse effects resulting from interactions between CAM therapies and conventional medications.

Lastly, the study revealed statistically significant differences in participants’ responses regarding reasons for CAM use according to whether they had ever used CAM themselves. These differences favored those with prior CAM experience, suggesting that CAM users showed higher levels of agreement with the scale items reflecting reasons that may lead MS patients to use CAM.

## Limitations of the study

This cross-sectional design precludes establishing causal relationships between CAM use and the associated factors, limiting the findings to observed associations at a single point in time. In addition, because the sample was restricted to patients with multiple sclerosis attending two neurology clinics in Riyadh and was not selected using random sampling, the generalizability of the results to all MS populations in Riyadh or other regions or countries may be limited.

This study has several additional limitations. First, data were obtained through a self-administered questionnaire, which may be subject to self-report and recall bias. Second, participation was voluntary, so selection bias cannot be completely ruled out, and the findings may not fully represent all eligible patients. Third, no formal a priori sample size calculation was performed; the sample size was instead determined pragmatically by the number of eligible patients attending the participating clinics during the study period, which means that some associations—particularly in smaller subgroups—may have been missed. Fourth, although the multi-dimensional reasons-for-use scale showed acceptable internal consistency, we did not conduct more advanced psychometric analyses, such as factor analysis or other assessments of construct validity; therefore, the construct validity of the scale remains to be established in future studies. Finally, adjusted multivariable analyses were not conducted because the sample size and distribution of covariates were insufficient to support a stable regression model; therefore, the observed associations should be interpreted as exploratory and hypothesis‑generating rather than confirmatory.

## Conclusion

The present study showed that the majority of patients with multiple sclerosis in Riyadh had used complementary and alternative medicine, with a clear preference for religious–spiritual modalities such as Islamic ruqyah and cupping therapy, alongside other practices including massage, oils, dietary supplements, honey, and herbal remedies. These findings highlight the central role that symptom relief, hope for a cure or for slowing disease progression, and socially driven recommendations (from relatives, healthcare providers, and the media) play in shaping patients’ decisions to use CAM. The results further revealed that educational level and marital status were significantly associated with CAM use, and that many patients did not disclose their CAM use to physicians or pharmacists, often because they did not perceive disclosure as important or were not asked about it. This finding suggests the potential importance of more proactive, non‑judgmental communication by healthcare providers regarding CAM, as well as educational interventions to raise patient awareness of potential interactions between CAM and conventional treatments.

Overall, the present study contributes to filling important knowledge gaps regarding patterns, reasons, and disclosure behaviors related to CAM use among MS patients in a Middle Eastern context, and it emphasizes the importance of integrating culturally sensitive discussions about CAM into routine clinical care. Future research using longitudinal designs and more diverse samples is warranted to further elucidate the impact of CAM use on clinical outcomes and to inform evidence‑based guidelines for the safe and coordinated use of CAM alongside conventional therapies. In addition, further social research with larger and more diverse samples is needed to provide an in‑depth analysis of the social determinants and factors that influence patients’ health behaviors related to CAM use. Moreover, additional studies in the field of medical sociology are required to analyse the relationship among physicians, pharmacists, and patients in managing the therapeutic process—including CAM—and to identify and address the obstacles encountered.

## Supplementary Information

Below is the link to the electronic supplementary material.

Supplementary file1 (PDF 277 KB)

## Data Availability

All the data associated with this study are included in the article.
