# Supplementary material for: Exploring the Landscape of Complementary and Alternative Medicine (CAM) among Multiple Sclerosis Patients in Riyadh: Types, Patterns, Health Implications, and Social Drivers – A Cross‑Sectional Study
Source: Saudi Pharm J. 2026 Jun 13;34(3):37. doi: 10.1007/s44446-026-00091-1 (PMC13264639; doi:10.1007/s44446-026-00091-1)
Supplement: Supplementary file 1 — Supplementary file1 (PDF 277 KB) [file 44446_2026_91_MOESM1_ESM.pdf]

استبيان دراسة علمية

استكشاف مشهد الطب التكميلي والبديل بين مرضى التصلب اللويحي المتعدد في مدينة الرياض

دراسة ميدانية في الأنواع، الاثار الصحية ، و الأسباب الاجتماعية للاستخدام.

عزيزي المشارك، عزيزتي المشاركة

شكرًا على موافقتك على المشاركة في هذا الاستبيان الالكتروني لدراسة بعنوان:

استكشاف مشهد الطب التكميلي والبديل بين مرضى التصلب اللويحي المتعدد في مدينة الرياض

- **علما أن بأنواع الطب التكميلي والبديل المشمول في هذه الدراسة هو**: الطب غير الدوائية المستخدمة من قبل المريض من دون وصفة طبية من قبل الطبيب مثل:

١. علاجات اليدوية (مثل: التدليك، الوخز بالإبر، تقويم العظام، الحجامة، الكي)

٢. الطب القائمة على المنتجات الطبيعية (مثل: الأعشاب، المكملات الغذائية، العلاج بمنتجات النحل)

٣. الأنظمة الغذائية الخاصة (مثل: الحمية الكيتونية، الحمية النباتية أو الخالية من الجلوتين)

والغرض من هذا الاستبيان الالكتروني هو: معرفة مدى استخدام الطب التكميلي والبديل والانواع المستخدمة لدى مرضى التصلب اللويحي المتعدد في المجتمع السعودي ، وتحديد الأسباب و الأثار، وسيستغرق اكمال هذا الاستبيان حوالي من ٥ إلى ١٠ دقيقة.

نؤكد لك أن جميع إجاباتك التي ستقدمها ستبقى في سرية تامة، وفي حال وجود أي استفسار لا تتردد في الاتصال بـ/ **د. تهاني الجهني** رقم الهاتف ٠١١٨٠٥٠٣٢٥أو **د. غادة الحمود** رقم الهاتف ٠١١٨٠٥٩٨٥ للإجابة على أسئلتك.

إذا كنت موافق على المشاركة في هذا الاستبيان الالكتروني فيرجى النقر على "التالي" للبدء.

الموقف من المشاركة في الاستبيان

- أوافق على المشاركة في هذا الاستبيان طواعية
- لا أوافق على المشاركة في هذا الاستبيان

إذا كنت موافق على المشاركة في هذا الاستبيان الالكتروني فيرجى النقر على "التالي" للبدء.

المحور الأول البيانات الأولية:

١-الجنس:

١-ذكر (   )

٢- انثى (   )

٢-العمر :

....

٣-المستوى التعليمي:

١-ثانوي فاقل (   )

٢-دبلوم (   )

٣-بكالريوس (   )

٤-ماجستير (   )

٥-دكتوراه (   )

٤-الحالة الاجتماعية:

١-عازب/ـة (   )

٢-متزوج/ـة (   )

٣-مطلقة/ـة (   )

٤-أرمل/ـة (   )

٥-هل تعمل ؟

١-نعم ( )

٢- لا ( )

٦-دخلك الشهري:

١-أقل من ٥٠٠٠ ريال ( )

٢-من ٥٠٠٠ إلى أقل من ١٠٠٠٠ ريال ( )

٣- من ١٠٠٠٠ ريال إلى أقل من ١٥٠٠٠ ريال ( )

٤-من ١٥٠٠٠ ريال فأكثر ( )

المحور الثاني الإجابة على تساؤلات الدراسة:

٧- بعد أن تم تشخيصك بالمرض، هل سبق أن جربت أيا من أنواع الطب التكميلي أو البديل؟

١-نعم ( )

٢-لا ( ) في حالة الإجابة "بـ لا " انتقل مباشرة إلى السؤال رقم ١٥

٨-في حال الإجابة "بنعم" أيا من أنواع الطب التكميلي والبديل التالية استخدمت: (يمكن اختيار أثر من إجابة)

١-الكي ( )

٢-لسع النحل ( )

٣-الحجامة ( )

٤-الإبر الصينية ( )

٥-التدليك ( )

٦-الرقية الشرعية ( )

٧-التأمل ( )

٨-اليوغا (   )

٩-العلاج بالموسيقى (   )

١٠-العلاج بالرسم (   )

١١-الأعشاب (   )

١٢-الزيوت (   )

١٣-المكملات لغذائية "دون وصفة من الطبيب" (   )

١٤-العلاج بالعسل (   )

١٥-الحمية النباتية (   )

١٦-الحمية الكيتونية (   )

١٧-الحمية الخالية من الجلوتين (   )

١٨-خلطات شعبية من العطارين (   )

١٩-أخرى، من فضلك أذكرها..........................

٩-هل سبق أن وجدت الفائدة المتوقعة من استخدامك للطب التكميلي و البديل؟

١-نعم عدة مرات (   )

٢-نعم مرة واحدة (   )

٣-لا لم يسبق أن حدث ذلك (   )

٤-أخرى ....

١٠- هل سبق وان حصلت لك اثار صحية سلبية نتيجة استخدام الطب التكميلي والبديل؟

١-نعم عدة مرات (   )

٢-نعم مرة واحدة (   )

٣-لا لم يسبق أن حدث ذلك (   )

٤-أخرى..........................

١١-هل سبق أن اخبرت طبيبك عن استخدامك للطب التكميلي و البديل؟

١-نعم ( )

٢-لا ( )

٣-أحيانا ( )

١٢-إذا لم تخبره ما هو السبب؟

١-لم يسألني الطبيب ( )

٢-لم أظن أن ذلك مهم ( )

٣-خشيت من النقد ( )

٤-أسباب أخرى، من فضلك أذكرها.....................................

١٣-هل سبق ان أخبرت الصيدلي عن استخدامك للطب التكميلي والبديل؟

١-نعم ( )

٢-لا ( )

٣-أحيانا ( )

١٤-إذا لم تخبره ما هو السبب؟

١-لم يسألني الصيدلي ( )

٢-لم أظن أن ذلك مهم ( )

٣-خشيت من النقد ( )

٤-أسباب أخرى، من فضلك أذكرها.....................................

١٥- في ما يلي عدد من العبارات حول الأسباب التي قد تؤدي إلى استخدام مرضى التصلب اللويحي للطب التكميلي والبديل ، نرجو وضع إشارة √ في الخانة التي تعبر عن درجة موافقتك على كل عبارة:

البعد	التسلسل	البند	لا أوافق بشدة	لا أوافق	أوافق إلى حد ما	أوافق	أوافق بشدة
الاعتقاد بأمان الطب التكميلي والبديلة	١	الاعتقاد بأن علاجات الطب التكميلي و البديل خالية من الأدوية الضارة.					
	٢	الاعتقاد بأن علاجات الطب التكميلي و البديل آمنة تمامًا عند استخدامها مع الأدوية الموصوفة					
	٣	الاعتقاد بأنه لا يوجد أي تعارض بين الأدوية الموصوفة والطب التكميلي والبديل.					
	٤	الاعتقاد بأن علاجات الطب التكميلي والبديل تعزز فعالية الأدوية الموصوفة.					
	٥	الاعتقادبأن علاجات الطب التكميلي و البديل يمكن استخدامها دون استشارة الطبيب.					

البعد الخاص بالعلاج الطبي	٦	شعور المريض بأن العلاج الطبي غير كافي لما يعاني منه المريض						
	٧	شعور المريض بأن العلاج الطبي غير مفيد لما يعاني منه.						
	٨	رغبة في التخفيف من الآثار الجانبية للعلاج الطبي						
البعد الخاص بالمرض	٩	رغبة المريض في التخلص من المرض						
	١٠	رغبة المريض في تخفيف الألم						
	١١	رغبة المريض في إبطاء تطور المرض						
البعد الخاص بتأثير المحيط الاجتماعي	١٢	يستخدم المريض الطب التكميلي والبديل إذا نصحه به أحد الأصدقاء						
	١٣	يستخدم المريض الطب التكميلي والبديل إذا نصحه به أحد أفراد الأسرة						

	١٤	يستخدم المريض الطب التكميلي والبديل إذا نصحه به أحد افراد الأقارب.					
	١٥	يستخدم المريض الطب التكميلي والبديل إذا نصحه به أحد المعارف (زميل عمل، جار)					
	١٦	يستخدم المريض الطب التكميلي والبديل إذا نصحه به مريض آخر بالتصلب اللويحي.					
	١٧	يستخدم المريض الطب التكميلي والبديل إذا نصحه به الصيدلي					
	١٨	تسويق بعض المحلات للعلاجات التكميلية والبديلة يؤدي إلى استخدمها من قبل المرضى					
البعد المعرفي	١٩	يعد قراءة المرضى عن علاجات الطب التكميلي والبديل في الكتب العلمية المتخصصة من أسباب استخدامهم لهم					
	٢٠	عرض فوائد الطب التكميلي والبديل في البرامج التلفازية يؤدي بالمريض الى استخدامها					

	٢١	ذكر فوائد أحد أنواع الطب التكميلي والبديل في المواقع الالكترونية يؤدي بالمريض إلى استخدامها					
	٢٢	تحدث أحد المؤثرين عبر في وسائل التواصل الاجتماعي عن فوائد أحد أنواع الطب التكميلي والبديل يؤدي بالمريض إلى استخدامه					

١٦–في حال كان هناك أسباب أخرى تدفع مرضى التصلب اللويحي المتعدد لاستخدام العلاج التكميلي أو البديل نرجو التكرم بذكرها:

....

شكرا لتعاونكم في الإجابة على أسئلة الاستبيان
